# The Association Between Surgical Site Infection and Prognosis of T4 Colorectal Cancer

**DOI:** 10.7759/cureus.66138

**Published:** 2024-08-04

**Authors:** Takuya Koike, Masaya Mukai, Kyoko Kishima, Daiki Yokoyama, Sayuri Hasegawa, Lin Fung Chan, Hideki Izumi, Kazutake Okada, Tomoko Sugiyama, Takuma Tajiri

**Affiliations:** 1 Department of Gastroenterological Surgery, Tokai University Hachioji Hospital, Hachioji, JPN; 2 Department of Pathology, Tokai University Hachioji Hospital, Hachioji, JPN

**Keywords:** relapse-free survival, overall survival, t4 colorectal cancer, prognosis, surgical site infection

## Abstract

Objectives: Patients with T4 colorectal cancer have poor prognosis, wherein no prognostic factors have been established. Surgical site infection (SSI) has been reported to be one of the risk factors for colorectal cancer recurrence. In this study, we evaluated the relationship between SSI occurrence and prognosis of T4 colorectal cancer and the prognostic impact of the site of SSI occurrence.

Methods: We examined 100 patients with T4 colorectal cancer who underwent radical surgery between April 2002 and December 2017, in a retrospective case-control study, excluding stage IV cases, and classified them into two groups: without SSI (non-SSI) and with SSI (SSI). The five-year relapse-free survival (RFS) and overall survival (OS) were calculated and compared between the two groups. The relationship between prognosis and the SSI site was also assessed according to the SSI site in the incisional/deep and organ/space SSI groups.

Results: The without SSI and with SSI groups included 73 and 27 patients, respectively. The five-year RFS was 55.1% and 22.2% in the without SSI and with SSI groups, respectively (hazard ratio (HR), 2.224; 95% confidence interval (CI), 1.269-3.898; *P*=0.005). The five-year OS was 67.0% and 38.4% in the without SSI and with SSI groups, respectively (HR, 2.366; 95% CI, 1.223-4.575; *P*=0.010). The patients in the with SSI group had a significantly poorer prognosis compared with the without SSI group. By SSI site, the prognosis was significantly worse in patients with SSI in the incisional/deep SSI group.

Conclusions: In T4 colorectal cancer, SSI occurrence was a high-risk factor for recurrence and may be a prognostic factor. This result suggested that patients with SSI occurrence may require close postoperative follow-up and appropriate adjuvant chemotherapy.

## Introduction

T4 colorectal cancer with local progression or invasion to other organs has a poor prognosis compared with colorectal cancer less than T4 [1]. Although various poor prognostic factors, such as tumor diameter, the influence of laparoscopic surgery, and SSI, have been reported, none have been established [2-6]. There are scattered reports indicating that the occurrence of surgical site infection (SSI) could be one of the poor prognostic factors after colorectal cancer surgery [7-13]. In addition, there are a few reports on the relationship between SSI and T4 colorectal cancer [6]. Therefore, in this study, we aimed to evaluate the relationship between the incidence of SSI and prognosis in patients with T4 colorectal cancer at our institution and the relationship between the site of SSI and prognosis.

## Materials and methods

Among patients with colorectal cancer who underwent radical surgery at our hospital between April 2004 and December 2017, data, collected from electronic medical records, of 100 T4 colorectal cancer cases, excluding stage IV cases of distant metastasis at the time of surgery, were retrospectively analyzed.

**Figure 1:**
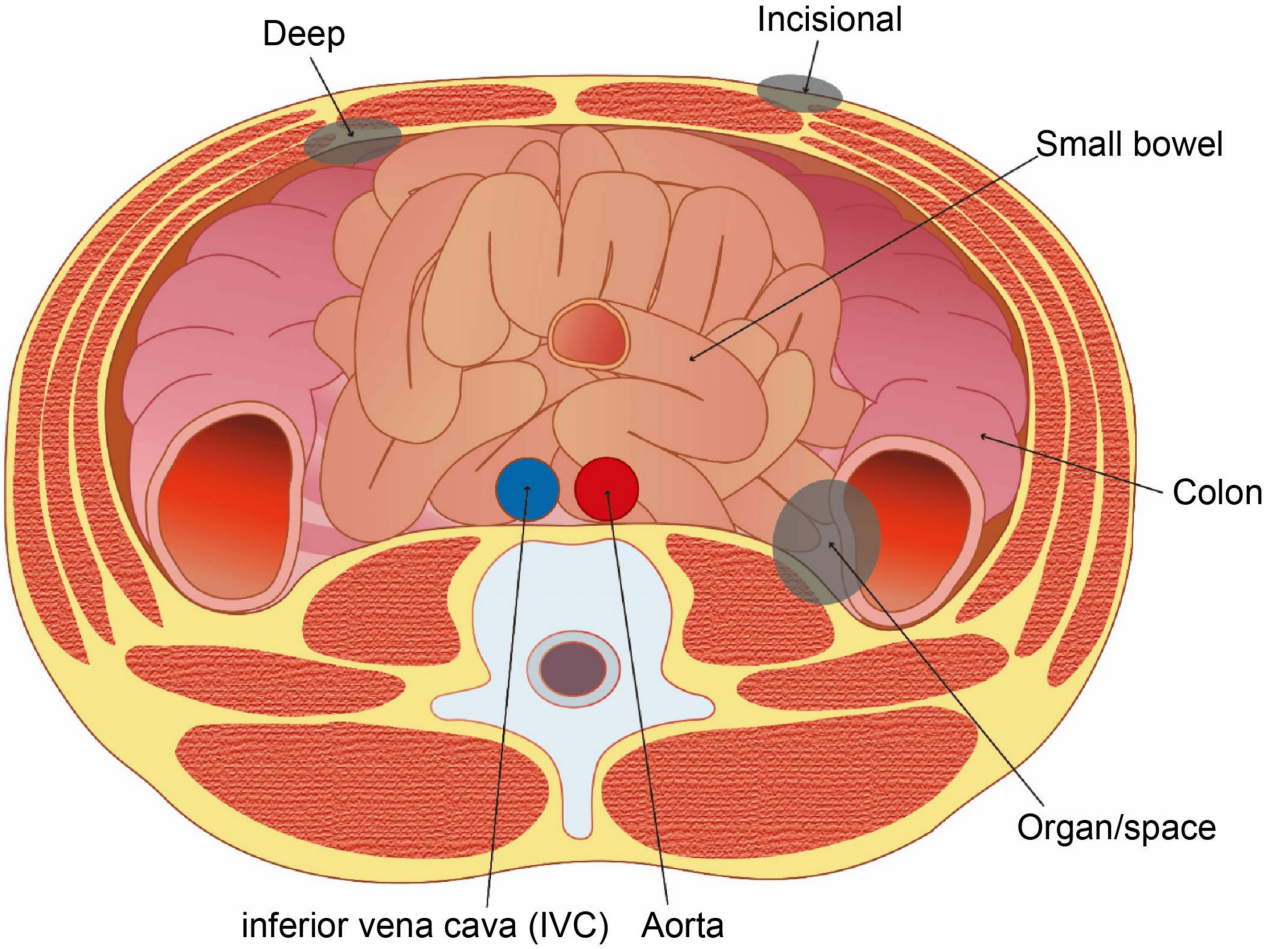
Schematic diagram of SSI on abdominal CT horizontal image SSI, surgical site infection

The patients were classified into two groups: without SSI (non-SSI) and with SSI (SSI). The five-year relapse-free survival (RFS) and overall survival (OS) rates were compared between these two groups. The SSI occurrence sites were classified into incisional/deep and organ/space SSI groups; the five-year RFS and OS rates were calculated among these three groups. The five-year RFS and OS were calculated for each pStage II and pStage III group, which were further classified into two groups: without SSI and with SSI. SSI was diagnosed according to the Japan Nosocomial Infections Surveillance (JANIS) protocol (Figure 1) [14].

The prophylactic antibacterial drugs were second-generation cephalosporins used only on the first and fourth postoperative day. The incisional closure method involved an interrupted suture with a synthetic absorbable thread for the fascia and a dermal-embedded suture with a synthetic absorbable thread or skinstapler for the subcutaneous area.

Postoperative adjuvant chemotherapy consisted of oral fluorouracil and intravenous irinotecan for six months to 2.5 years, followed by surgery or intense chemotherapy, including oxaliplatin, at the time of recurrence.

The calculated RFS and OS rates of the patients were based on the data extracted from the electronic medical and pathology records of Hachioji Hospital of Tokai University School of Medicine. Recurrence was defined as the date when recurrence was confirmed by CT or magnetic resonance imaging, starting from the date of surgery. Death date was the date of death from any cause. The patients who were transferred to other hospitals during the course of the disease were followed up after confirming their transfer (follow-up rate: 87.0%).

Statistical analyses

The five-year RFS and OS rates were calculated using the Kaplan-Meier method. The hazard ratio (HR) and 95% confidence interval (CI) were evaluated using the Cox proportional hazards regression model. The risk factors for five-year RFS and five-year OS risk factors were evaluated by univariate and multivariate analyses using Cox regression models. Factors that were significant from the univariate analysis were fitted in the multivariate analysis of the five-year RFS and five-year OS. Results of the Cox regression model are presented as HR and 95% CI. Regarding the analysis of patient background: age; body mass index; tumor length and diameter; operative time; and blood loss, the nonparametric Mann-Whitney U test was applied. However, sex, smoking history, diabetes mellitus incidence, American Society of Anesthesiologists physical status classification, lesion site, depth, pStage, procedure, and chemotherapy were analyzed using the chi-square or Fisher’s exact test. A p-value <0.05 was considered statistically significant. Statistical analysis was performed using IBM SPSS Statistics for Windows Version 28.0 (IBM Corp., Armonk, NY, USA).

This study was conducted in accordance with the Declaration of Helsinki (as revised in 2013). This study was approved by the appropriate Institutional Review Board (details blinded for peer review). Written informed consent was obtained from all patients.

## Results

**Figure 2:**
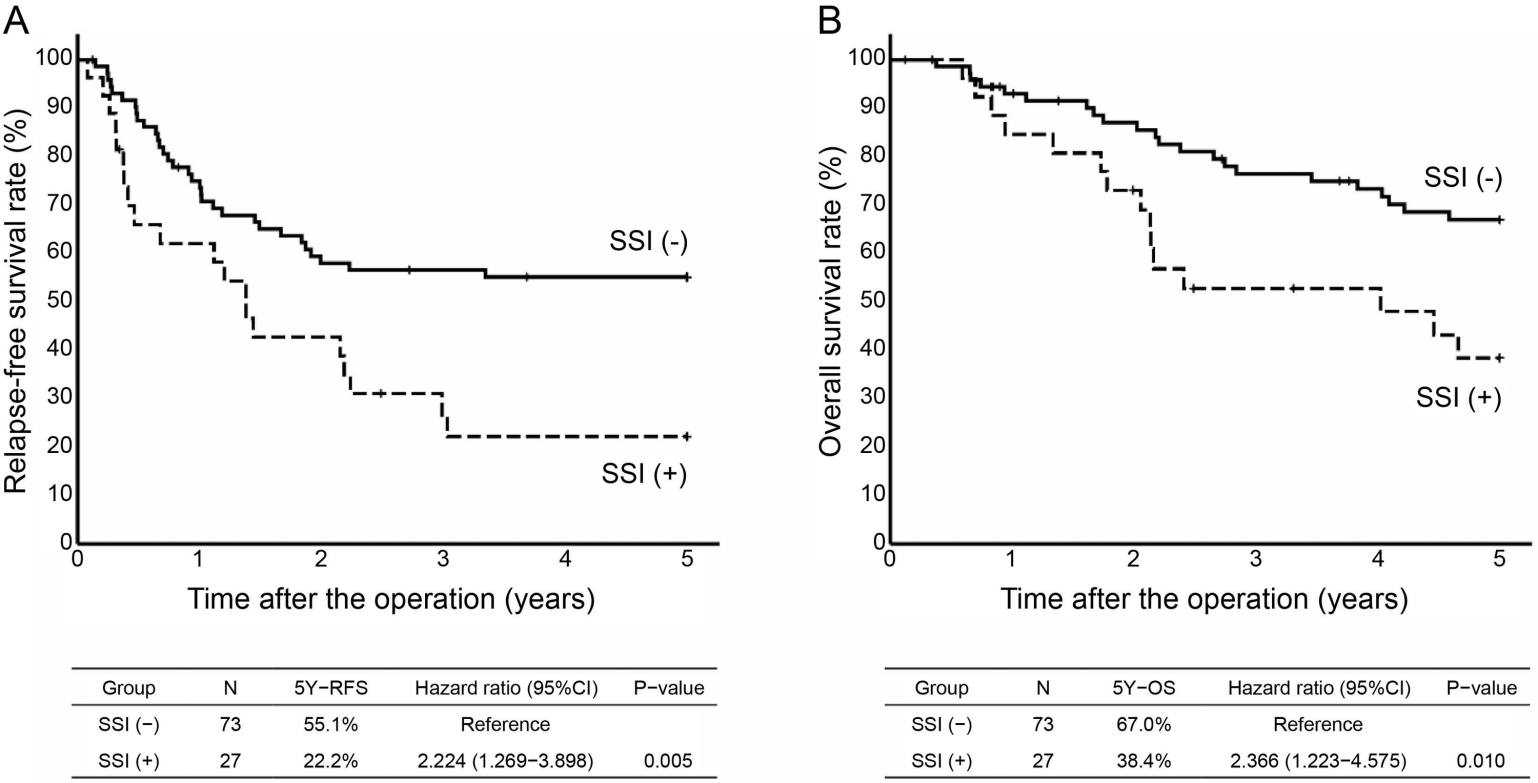
A. Kaplan-Meyer curves for five-year RFS by two groups (without SSI and with SSI). B. Kaplan-Meyer curves for five-year OS by two groups (without SSI and with SSI) SSI, surgical site infection; RFS, relapse-free survival; OS, overall survival

The without SSI and with SSI groups included 73 and 27 patients, respectively. The five-year RFS was 55.1% and 22.2% in the without SSI and with SSI groups, respectively (HR, 2.224; 95% CI, 1.269-3.898; P=0.005). The five-year OS in the without SSI and with SSI groups was 67.0% and 38.4%, respectively (HR, 2.366; 95% CI, 1.223-4.575; P=0.010). The with SSI group had significantly worse five-year RFS and OS compared with the without SSI group (Figure 2). There were no significant differences in the factors known to influence the occurrence of SSI between the two groups (Table 1). Regarding the five-year RFS, univariate analysis showed that SSI occurrence, smoking history, blood loss, and pStage were associated with SSI occurrence (Table 2). Regarding the five-year OS, univariate analysis showed that SSI, smoking history, blood loss, pStage, and operative approach were associated (Table 3). This association was maintained even after performing multivariate analysis; SSI occurrence was a poor prognostic factor (Tables 2, 3).

**Table 1:** The characteristics of 100 patients with colorectal cancer surgery *ASA physical status classification **Japanese classification of colorectal carcinoma (ninth edition) BMI, body mass index; HALS, hand-assisted laparoscopic surgery; SSI, surgical site infection; ASA, American Society of Anesthesiologists

Variable	Total (n=100)	SSI (-) (n=73)	SSI (+) (n=27)	p-value
Gender, n				0.742
Male	51	36	15	
Female	49	37	12	
Age, median (range), year	71 (35-91)	71 (35-88)	69 (40-91)	0.365
BMI, median (range), kg/m^2^	21.5 (14.7-35.2)	21.6 (14.7-35.2)	20.4 (15.2-32.5)	0.387
Smoking, n				
Non-smoker	54	39	15	>0.999
Smoker	46	34	12	
Diabetes, n				0.224
No	85	64	21	
Yes	15	9	6	
ASA score* (n)				0.771
Class I	5	3	2	
Class Il	74	54	20	
Class Ill	21	16	5	
Tumor site, n				>0.999
Colon	72	53	19	
Rectum	28	20	8	
Depth**, n				0.739
T4a	71	53	18	
T4b	29	20	9	
Tumor length, median (range), mm	50 (15-140)	50 (18-140)	50 (15-120)	0.188
pStage**, n				0.414
pStage Il	38	30	8	
pStage Ill	62	43	19	
Operative approach, n				0.386
Open	54	37	17	
HALS/laparoscopic	46	36	10	
Operation time, median (range), minutes	192 (95-545)	186 (95-406)	218 (107-545)	0.1 19
Blood loss, median (range), mL	241 (0-10278)	200 (0-2616)	388 (0-10278)	0.032
Adjuvant chemotherapy, n				0.499
No	33	26	7	
Yes	67	47	20	

**Table 2:** Prognostic analysis of five-year RFS in patients with colorectal cancer *ASA physical status classification **Japanese classification of colorectal carcinoma (ninth edition) BMI, body mass index; HALS, hand-assisted laparoscopic surgery; SSI, surgical site infection; ASA, American Society of Anesthesiologists; RFS, relapse-free survival

Variable	Univariable		Multivariable	
	Hazard ratio (95% CI)	P-value	Hazard ratio (95% CI)	P-value
Gender, n				
Male	1			
Female	1.442 (0.834-2.494)	0.19		
Age, median (range), year	1.011 (0.987-1.036)	0.376		
BMI, median (range), kg/m^2^	0.998 (0.925-1.077)	0.966		
Smoking, n				
Non-smoker	1		1	
Smoker	0.541 (0.307-0.952)	0.033	0.510 (0.286-0.908)	0.022
Diabetes, n				
No	1			
Yes	1.021 (0.481-2.170)	0.956		
ASA score*, n				
Class I	1			
Class II	0.512 (0.182-1.437)	0.203		
Class III	0.511 (0.160-1.633)	0.258		
Tumor site, n				
Colon	1			
Rectum	0.519 (0.260-1.035)	0.063		
Depth**, n				
T4a	1			
T4b	1.026 (0.563-1.871)	0.933		
Tumor length, median (range), mm	1.009 (0.998-1.019)	0.096		
pStage**, n				
pStage II	1		1	
pStage III	2.070 (1.120-3.829)	0.02	2.073 (1.118-3.844)	0.021
Operative approach, n				
Open	1			
HALS/ Laparoscopic	0.601 (0.345-1.047)	0.072		
Operation time, median (range), minutes	1.002 (0.999-1.005)	0.215		
Blood loss, median (range), mL	1.000 (1.000-1.000)	0.036	1.000 (1.000-1.000)	0.056
Adjuvant chemotherapy, n				
No	1			
Yes	0.815 (0.460-1.444)	0.486		
SSI, n				
SSI (-)	1		1	
SSI (+)	2.224 (1.269-3.898)	0.005	2.016 (1.138-3.572)	0.016

**Table 3:** Prognostic analysis of five-year OS in patients with colorectal cancer *ASA physical status classification **Japanese classification of colorectal carcinoma (ninth edition) BMI, body mass index; HALS, hand-assisted laparoscopic surgery; SSI, surgical site infection; ASA, American Society of Anesthesiologists; RFS, relapse-free survival; OS, overall survival

Variable	Univariable		Multivariable	
	Hazard ratio (95% CI)	P-value	Hazard ratio (95% CI)	P-value
Gender, n				
Male	1			
Female	1.600 (0.830-3.084)	0.161		
Age, median (range), year	1.030 (0.999-1.063)	0.061		
BMI, median (range), kg/m^2^	0.994 (0.910-1.085)	0.886		
Smoking, n				
Non-smoker	1		1	
Smoker	0.356 (0.172-0.737)	0.005	0.401 (0.191-0.842)	0.016
Diabetes, n				
No	1			
Yes	0.755 (0.267-2.135)	0.597		
ASA score*, n				
Class I	1			
Class II	0.367 (0.127-1.054)	0.063		
Class III	0.329 (0.092-1.176)	0.087		
Tumor site, n				
Colon	1			
Rectum	0.550 (0.242-1.253)	0.155		
Depth**, n				
T4a	1			
T4b	1.326 (0.675-2.606)	0.412		
Tumor length, median (range), mm	1.006 (0.995-1.017)	0.288		
pStage**, n				
pStage II	1		1	
pStage III	2.337 (1.101-4.958)	0.027	3.043 (1.412-6.558)	0.005
Operative approach, n				
Open	1		1	
HALS/laparoscopic	0.298 (0.140-0.632)	0.002	0.286 (0.132-0.621)	0.002
Operation time, median (range), minutes	1.002 (0.998-1.006)	0.336		
Blood loss, median (range), mL	1.000 (1.000-1.000)	0.032		
Adjuvant chemotherapy, n				
No	1			
Yes	0.521 (0.270-1.006)	0.052		
SSI, n				
SSI (-)	1		1	
SSI (+)	2.366 (1.223-4.575)	0.01	2.189 (1.125-4.260)	0.021

**Figure 3:**
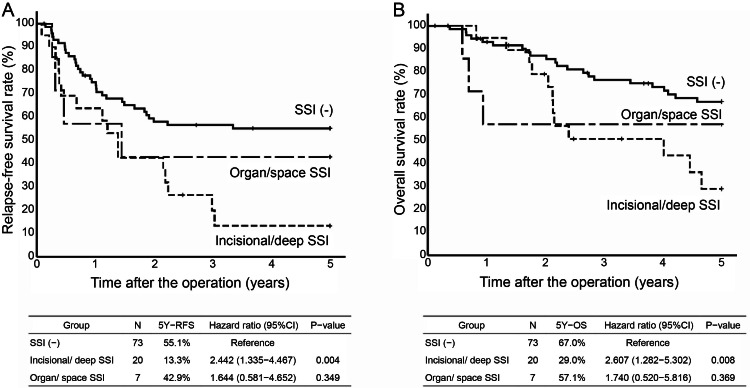
A. Kaplan-Meyer curves for five-year RFS by three groups (without SSI, SSI (incisional/deep), and SSI (organ/space)). B. Kaplan-Meyer curves for five-year OS by three groups (without SSI, SSI, (incisional/deep) and SSI (organ/space)) SSI, surgical site infection RFS, relapse-free survival OS, overall survival

The with SSI group was further divided into incisional/deep SSI and organ/space SSI groups. Twenty cases were classified into the incisional/deep SSI group and seven into the organ/space SSI group. The five-year RFS was 13.3% in the incisional/deep SSI (HR, 2.442; 95% CI, 1.335-4.467; P=0.004) and 42.9% in the organ/space SSI groups (HR, 1.644; 95% CI, 0.581-4.652; P=0.349). The five-year OS was 29.0% in the incisional/deep SSI (HR, 2.607; 95% CI, 1.282-5.302; P=0.008) and 57.1% in the organ/space SSI groups (HR, 1.740; 95% CI, 0.520-5.816; P=0.369), with significant differences in incisional/deep SSI (Figure 3).

**Figure 4:**
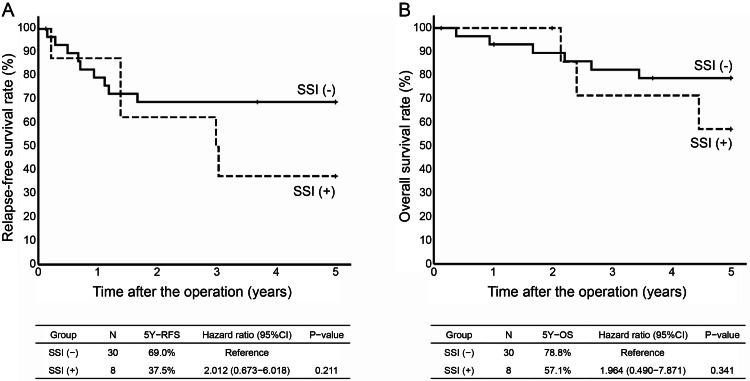
A. Kaplan-Meyer curves for five-year RFS by two groups (without SSI and with SSI) in patients with pStage Ⅱ. B. Kaplan-Meyer curves for five-year OS by two groups (without SSI and with SSI) in patients with pStage Ⅱ SSI, Surgical site infection RFS, relapse-free survival OS, overall survival

**Figure 5:**
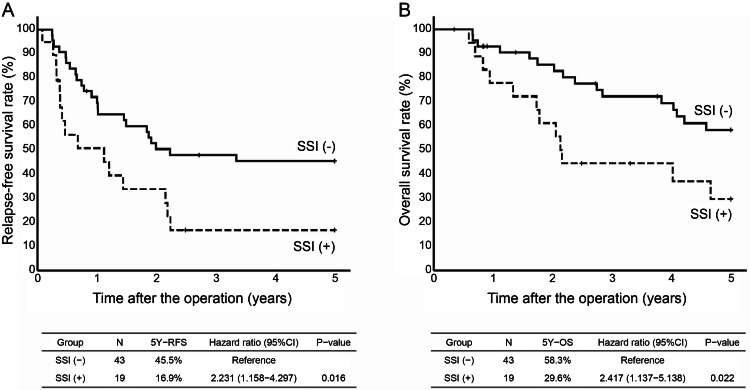
A. Kaplan-Meyer curves for five-year RFS by two groups (without SSI and with SSI) in patients with pStage Ⅲ. B. Kaplan-Meyer curves for five-year OS by two groups (without SSI and with SSI) in patients with pStage Ⅲ SSI, surgical site infection; RFS, relapse-free survival; OS, overall survival

Considering the examination of each stage, pStage II was observed in 38 cases, which were classified as 30 cases in the without SSI group and eight cases in the with SSI group. The five-year RFS in the without SSI group was 69.0% and in the with SSI group was 37.5% (HR, 2.012; 95% CI, 0.673-6.018; P=0.211). The five-year OS in the without SSI group was 78.8% and in the with SSI group was 57.5% (HR, 2.012; 95% CI, 0.673-6.018; P=0.211). The five-year OS in the without SSI group was 78.8% and in the with SSI group was 57.1% (HR, 1.964; 95% CI, 0.490-7.871; P=0.341) (Figure 4). pStage III was observed in 62 patients (43 in the without SSI group and 19 in the with SSI group). The five-year RFS was 45.5% in the without SSI and 16.9% in the with SSI groups (HR, 2.231; 95% CI, 1.158-4.297; P=0.016). The five-year OS in the without SSI group was 58.3% and in the with SSI group was 29.6% (HR, 2.417; 95% CI, 1.137-5.138; P=0.022), the with SSI group had a significantly worse prognosis for both five-year RFS and OS (Figure 5).

## Discussion

Various poor prognostic factors for T4 colorectal cancer have been reported, including a relationship with tumor diameter and the influence of laparoscopic surgery [2-5]. However, no established poor prognostic factors have been recognized.

In colorectal cancer, organ/space SSIs are often attributed to suture failure and have been reported to be a risk factor for postoperative recurrence [7]. The reasons for this include delayed postoperative oral intake and the need for urgent reoperation, such as colostomy, which may lead to deterioration in nutritional status; postoperative immunocompromised state, which may influence recurrence; and delayed induction of adjuvant chemotherapy in patients with advanced cancer [15,16]. In the present study, there were only seven cases of organ/space SSI, without showing statistically significant differences. This may be due to the long observation period and the fact that Hartmann's surgery, which does not require anastomotic manipulation, is often performed for locally advanced cancer in early cases. Hartmann's surgery was performed in cases where the attending physician determined that the patient was in poor general condition. The use of a colostomy allowed oral intake in the early postoperative period, and the risk of suture failure was almost zero, which may have reduced the incidence of SSI and may have been advantageous in maintaining immunity in the postoperative period.

In this study, incisional/deep SSI, which is less likely to affect systemic status, was associated with significantly poorer outcomes regarding both the five-year RFS and OS rates. This suggests that immune system compromise may be involved in the occurrence of incisional/deep SSI. In regard to the analysis of prognostic factors, smoking history, pStage, and blood loss were associated with a poor prognosis, in addition to SSI occurrence. Among these, smoking history and blood loss are recognized as factors that could cause SSI and are thought to influence the weakening of immunity [8]. We suggest that patients with SSI are immunocompromised to some extent; additional studies are needed to assess the relationship between nutritional status and cancer immunity [15,16]. To assess the risk of these biases, we selected 18 patients in each group without SSI and with SSI by propensity score matching using gender, age, BMI, smoking history, diabetes, ASA, tumor length, pStage, operative approach, operation time, and blood loss as confounding factors. The with SSI group had a worse poor prognosis in both 5YRFS and 5YOS (data not shown). In addition, the incidence of SSI after colorectal cancer surgery in Japan has been reported to be approximately 10-15% [14]. However, in this study, a relatively high incidence of SSI (27.0%) was found. Although this study was conducted including T4 cases of local progression, which are considered to have a high incidence of SSI, to improve the high SSI incidence rate, perioperative management was reviewed, and SSI surveillance was started in April 2023. We plan to examine whether this would reduce the incidence of SSIs and improve prognosis.

In the analysis of stage events, a trend toward a poor prognosis was found in the group with SSIs in both pStage II and pStage III, with a significant difference in patients with pStage III. The Japanese guidelines recommend postoperative adjuvant chemotherapy for pStage II in patients at high risk (with T4, vascular invasion, poorly differentiated carcinoma, perforation, and bowel obstruction) [17]. Moreover, the occurrence of SSI in addition to T4 may help determine whether such therapy is indicated. In addition, six months of postoperative adjuvant chemotherapy is recommended for pStage III cases. However, although the efficacy of adding L-OHP is recommended, decision-making could be challenging due to side effects such as age and residual neurological deficits after chemotherapy. Therefore, in many cases, deciding whether to perform the procedure is difficult. Similar to pStage II, the occurrence of SSI is expected to be the basis for determining the addition of L-OHP as a chemotherapeutic agent.

An advantage of using SSI occurrence as a risk factor for recurrence is that no additional postoperative examinations or procedures are required. In Japan, since the primary surgeon generally performs daily postoperative rounds, this method may help identify high-risk groups for recurrence with only routine postoperative management without additional costs.

This study has several limitations. First, the sample size is small. Second, the findings cannot be generalized to other populations. Third, the closing method was not standardized due to the long observation period. Fourth, we must also consider the possibility that smoking history, pStage, and blood loss are confounding factors. To resolve these problems, a multicenter study with a uniform closure method and observation period is required.

The with SSI group classified in this study was a factor in identifying cases of poor prognosis and may be one of the reasons for conducting strict postoperative observation and adjuvant chemotherapy. The with SSI group was considered a rationale for aggressively introducing chemotherapy in patients with pStage II, with little consensus on the use of adjuvant chemotherapy in patients with pStage III wherein the decision whether to receive intravenous anticancer agents, such as L-OHP, was unsure.

## Conclusions

Our study indicates that the occurrence of SSI in patients with T4 colorectal cancer is a high-risk factor for recurrence and may be a prognostic factor. However, there are some points to be considered, such as the small number of samples and non-standardized closing methods. Further study is needed to increase the number of samples in the future.
